# Genome Sequence of Bacillus subtilis natto VK161, a Novel Strain That Produces Vitamin K_2_

**DOI:** 10.1128/MRA.00444-19

**Published:** 2019-08-29

**Authors:** Dylan Parks, In-Hwa Chung, In kyu Lee, Eui-Joong Kim, Sarbjeet Niraula, Woo-Suk Chang

**Affiliations:** a Department of Biology, University of Texas, Arlington, Texas, USA; b SungWun Pharmacopia/Bio Co., Ltd., Seoul, Republic of Korea; c GF Fermentech, Inc., Sejong, Republic of Korea; University of Arizona

## Abstract

Bacillus subtilis strain natto VK161 was selected for its high production of vitamin K_2_. Its genome was sequenced and annotated in the Department of Energy-Joint Genome Institute (DOE-JGI) annotation pipeline. It resulted in a chromosome of 4,073,396 bp, which is composed of 4,332 protein-coding genes, 23 rRNA genes, and 77 tRNA genes.

## ANNOUNCEMENT

Bacillus subtilis natto is a Gram-positive, aerobic, spore-forming bacterium closely related to the laboratory strain B. subtilis Marburg 168, which was the first sequenced genome of the B. subtilis family (1). Although there are several natto strains, only the B. subtilis natto BEST 195 strain has been sequenced completely (2). B. subtilis natto is known to produce poly-γ-glutamate (γ-PGA), which gives a characteristic slimy texture to the fermented soybean product called natto (3). In addition, its potential applications in therapeutics (4) and probiotics (5) have been reported in the scientific community.

Bacillus subtilis natto VK161 was selected for its high production of menaquinone, known as vitamin K_2_, using chemical mutagenesis with *N*-methyl-*N′*-nitro-*N*-nitrosoguanidine (NTG). Briefly, the parental strain B. subtilis natto KCCM 12027 obtained from the Korean Culture Center of Microorganisms (KCCM) was preexposed to NTG. We screened for vitamin K_2_ production through the NTG-induced mutagenesis library and found that a single mutant colony, subsequently named B. subtilis natto VK161, produced the highest level of vitamin K_2_ (ca. 120 mg/liter). Here, we report the genome sequence of B. subtilis natto VK161. Strain VK161 was cultured in Luria-Bertani (LB) medium overnight at 37°C with shaking at 200 rpm. Genomic DNA (gDNA) of B. subtilis natto VK161 was isolated using the G-spin genomic extraction kit (iNtRON Biotechnology). The amount of gDNA (>250 ng/μl) and its quality (260/280 ratio, ≈1.8) were determined using a NanoDrop spectrophotometer (Thermo Fisher Scientific). Gel electrophoresis was also used to inspect gDNA, confirming that a band was above 10 kb of a DNA ladder marker. Sequencing was performed at the University of Texas at the Austin Genomic Sequencing and Analysis Facility (GSAF) on an Illumina MiSeq platform with paired-end (PE) 2 × 300-bp run specifications. Sequencing produced 2,260,886 reads for approximately 170× coverage. Raw reads were quality filtered, and adapter sequences were subsequently removed using BBDuk, one of the BBTools developed at the U.S. Department of Energy-Joint Genome Institute (DOE-JGI), using the parameters ktrim=r, k = 23, mink = 11, hdist = 1, qtrim=r, trimq = 10, ftm = 5, maq = 25, and minlen = 100. Filtered read quality was ensured by inspection using FastQC (6). Default parameters were used for all other software programs, unless otherwise specified. Assembly of filtered reads was performed using SPAdes 3.10.1 (7) and assembly quality assessed via QUAST 4.6.1 (8). The genome assembly contained 96 contigs (≥1,000 bp) totaling 4.1 Mbp, with an *N*_50_ value of 78,653 bp.

Functional annotation and gene prediction were performed using the DOE-JGI Microbial Genome Annotation Pipeline (9). JGI’s Integrated Microbial Genomes (IMG) system (10) showed that the complete genome of strain VK161 consists of 4,073,396 bp, with 4,332 coding genes (63.9% with predicted functions), 23 rRNA genes, 77 tRNA genes, and an average G+C content of 43.33%. Further annotation and gene prediction were performed using JGI’s IMG-Expert Review (IMG ER) software (11).

### Data availability.

The whole-genome shotgun project of B. subtilis natto VK161 has been deposited into NCBI GenBank under the accession number SJSU00000000. The version described in this paper is the first version, SJSU01000000. Raw sequences were deposited in the NCBI SRA database under the accession number SRP192779.
